# An Overview of Lipid Metabolism and Nonalcoholic Fatty Liver Disease

**DOI:** 10.1155/2020/4020249

**Published:** 2020-07-18

**Authors:** Ke Pei, Ting Gui, Dongfang Kan, Huichao Feng, Yanqiang Jin, Ying Yang, Qian Zhang, Ziwei Du, Zhibo Gai, Jibiao Wu, Yunlun Li

**Affiliations:** ^1^College of Traditional Chinese Medicine, Shandong University of Traditional Chinese Medicine, Jinan, China; ^2^Experimental Center, Shandong University of Traditional Chinese Medicine, Jinan, China; ^3^Key Laboratory of Traditional Chinese Medicine Classical Theory, Ministry of Education, Shandong University of Traditional Chinese Medicine, Jinan, China; ^4^Shandong Provincial Key Laboratory of Traditional Chinese Medicine for Basic research, Shandong University of Traditional Chinese Medicine, Jinan, China; ^5^Acupuncture and Massage College, Shandong University of Traditional Chinese Medicine, Jinan, China; ^6^Traditional Chinese Medicine Department, Tai'an Maternal and Child Health Hospital, Tai'an, China; ^7^First Clinical Medical College, Shandong University of Traditional Chinese Medicine, Jinan, China; ^8^Institute of Rehabilitation, Shandong University of Traditional Chinese Medicine, Jinan, China; ^9^Department of Clinical Pharmacology and Toxicology, University Hospital Zurich, University of Zurich, 8006 Zurich, Switzerland; ^10^The Affiliated Hospital of Shandong University of Traditional Chinese Medicine, Jinan, China

## Abstract

The occurrence of nonalcoholic fatty liver disease (NAFLD) is associated with major abnormalities of hepatic lipid metabolism. We propose that lipid abnormalities directly or indirectly contribute to NAFLD, especially fatty acid accumulation, arachidonic acid metabolic disturbance, and ceramide overload. The effects of lipid intake and accumulation on NAFLD and NAFLD treatment are explained with theoretical and experimental details. Overall, these findings provide further understanding of lipid metabolism in NAFLD and may lead to novel therapies.

## 1. Introduction

Nonalcoholic fatty liver disease (NAFLD) is one of the most common liver diseases in Western countries, and its prevalence is increasing worldwide [1, 2]. Recently, the prevalence of NAFLD was reported to be higher in South America and Asia than in Europe and USA [3]. The spectrum of NAFLD ranges from simple steatosis to nonalcoholic steatohepatitis (NASH), fibrosis, cirrhosis, and hepatocellular carcinoma (HCC), which is caused by excessive caloric intake without excessive alcohol consumption [4]. Fatty liver development without heavy drinking is generally called nonalcoholic fatty liver (NAFL) [5]. NASH is the intensified form of NAFL, which is associated with inflammation and different degrees of fibrosis [5, 6]. Nearly 10%–25% of patients with NASH may develop cirrhosis [7]. However, in some patients, NASH can progress to hepatocellular carcinoma without significant cirrhosis [8]. NASH is projected to be the leading cause of liver transplantation in USA by 2020, which is due to increasing disease prevalence and ineffective treatment [9].

Recent studies on the relationship between NAFLD and lipid disorders have demonstrated that disruption of the lipid metabolism balance in the liver causes lipid accumulation and consequently, hepatotoxicity, and NAFLD [10–12]. Dyslipidemia manifests as an increase in plasma free fatty acids (FFA), oxidized low-density lipoprotein (ox-LDL), and triglycerides (TGs), which cause inflammation, oxidative stress, lipotoxicity, and liver damage [11, 12]. Dyslipidemia can occur at all stages of NAFLD and aggravate the NAFLD process. Few studies have emphasized on the role of ceramide and arachidonic acid metabolism in the development of NAFLD. Therefore, we reviewed the metabolic pathways contributing to the pathogenesis of NAFLD by elaborating the role of fatty acid (FA), ceramide, and arachidonic acid as well as studying lipid uptake and potential treatments.

## 2. Factors Influencing NAFLD

Weight gain and obesity are primary risk factors for the development of fatty liver. Further evidence suggests that diet composition, particularly carbohydrates, has an important role in the progression of disease to NASH and fibrosis [6].

### 2.1. Obesity and NAFLD

Obesity refers to the excessive accumulation of body fat due to the imbalance of cellular lipogenic and lipolytic activities [13]. The incidence of obesity has been increasing worldwide for the past two decades; the prevalence in South America, Middle East, Asia, the United States, and Europe was reported to be 31%, 32%, 27%, 24%, and 23%, respectively [14–16]. In addition, the morbidity of NAFLD is increasing [17]. Although the awareness emphasis on healthy lifestyle is increasing, obesity remains a public health problem, leading to many preventable complications such as NAFLD [5]. A study indicated that 80% of NAFLD patients are obese (body mass index (BMI) > 30 kg/m^2^) as well as the large amount of visceral adipose tissue (VAT) in morbidly obese (BMI > 40 kg/m^2^) individuals which leads to high morbidity of NAFLD [18]. Ciupińska-Kajor et al. reported that morbid obesity is associated with a higher incidence of more advanced fibrosis and confirmed that severe fibrosis and cirrhosis are common to a greater extent in morbidly obese patients with NAFLD [19]. Therefore, NAFLD treatments are focused on weight loss through lifestyle changes, antiobesity drugs, and bariatric surgery [20].

### 2.2. High-Carbohydrate Diet Intake and NAFLD

Excessive carbohydrate intake is closely associated with the occurrence of NAFLD. It has been demonstrated that a high-carbohydrate diet pattern characterized by high intake of fruits, cakes, ice cream, soft drinks, and candied fruits is positively associated with the prevalence of NAFLD [21]. The higher risk of NAFLD due to fruits, sugared beverages, and snacks may be because these foods contain large amounts of sugars such as fructose and sucrose that have been associated with the pathophysiology of NAFLD. Among the three most important carbohydrates (glucose, fructose, and sucrose), fructose and sucrose have some influence on the epidemiology of NAFLD. Fructose is the main component of sweeteners, and its intake has tripled over the past decade [22]. It can cause liver damage through a variety of mechanisms [23]. In fact, several epidemiological and experimental studies have supported the potential pathogenic effects of increased fructose consumption [24, 25]. Different animal models have been studied to explore the mechanism of fructose-induced NAFLD. In humans, excess fructose intake is associated with elevated plasma TG level and hepatic lipid deposition [26]. A study demonstrated that high-calorie foods (65% sucrose foods) for 8 weeks resulted in obesity, insulin resistance, and macrovesicular steatosis in C57BL/6 mice [27]. Hence, targeting excessive fructose and sucrose consumption that cause NAFLD, dietary guidelines have recommended to limit added sugars (primarily sucrose and high-fructose corn syrup (HFCS)) in the diet to a maximum 5%–10% of daily calories [28, 29].

## 3. Lipid Uptake in the Normal Liver and in NAFLD

The process of lipid uptake in the liver in both physiological and pathological conditions is mediated through liver FA binding protein (FABP1) and CD36. Abnormal protein regulation may lead to excessive hepatic accumulation of nonesterified FAs (NEFAs) and TG, causing cytotoxicity and resulting in NAFLD.

### 3.1. CD36-Mediated Lipid Uptake

CD36 is the major receptor involved in long-chain FA transport and TG storage and is expressed in several cells such as macrophages and monocytes [30] and tissues such as the liver, heart, and adipose [31]. It recognizes modified lipoproteins such as ox-LDL, promotes lipid-laden foam cell formation [32], and modulates events associated with lipid utilization. Recently, CD36 was found to play a crucial role in the liver by participating in FA uptake and storage and secretion of TG [33]. CD36 mediates FFA uptake in various tissues, and FA uptake has a significant role in hepatic steatosis; thus, abnormalities in CD36 may lead to hepatic steatosis [34]. The majority of NEFAs in the blood are bound to carrier proteins (mainly albumin), and their uptake requires dissociation from CD36-mediated carrier proteins [35]. Excessive hepatic accumulation of NEFAs and TG leads to cytotoxicity, resulting in NAFLD progression. Li et al. indicated that CD36 is the negative regulator of lipophagy in liver cells [36]. It has been reported that CD36 expression is significantly upregulated in hepatic tissues of NAFLD patients [37] and overexpression of CD36 in the liver increases TG accumulation and causes hepatic steatosis progression [38]. The localization of CD36 on the plasma membrane of liver cells was significantly higher in NASH patients than that in patients with normal liver and those with simple steatosis [39]. In the livers of mice with NASH, increased CD36 palmitoylation and CD36 localization on the plasma membrane of hepatocytes were observed. In addition, inhibition of CD36 palmitoylation protected mice from developing NASH [39]. Briefly, CD36 expression is increased in the liver of NAFLD patients, which leads to abnormal liver function and systemic abnormalities, including inflammation, hepatocyte damage, hepatic lipid accumulation, and fibrosis.

### 3.2. FABP1-Mediated Lipid Uptake

FABP1 or L-FABP is the first discovered member of the FABP family, with high concentrations in the liver, intestine, and kidney. In addition to CD36, FABP1 mediates the uptake, transport, and metabolism of long-chain FAs and other lipid ligands in cells [40]. It is a soluble protein commonly found in rodents (26% cytosolic protein; 200–400 *μ*M) and humans (7%–10% cytosolic protein; 700–1,000 *μ*M). Recent studies using cell and mice models have verified that FABP1 is the key regulator of lipid metabolism and steatosis in the liver [41]. In vitro studies revealed that FA uptake was significantly increased with FABP1 overexpression and was significantly decreased with FABP1 antisense ribonucleic acid (RNA) expression [42]. A Western diet of high saturated fat and high cholesterol could prevent diet-induced obesity and hepatic steatosis in FABP1^−/−^ mice, which reflected changes in the kinetics of saturated FA (SFA) utilization [42]. Human genetic variant of *L-FABP* gene is linked to abnormal lipid metabolism [42]. Studies have indicated that FFA-induced hepatic steatosis and liver injury can be improved by inhibiting FABP1 expression [41]. Downregulation of FABP1 has been identified as the new mechanism for preventing hepatic steatosis and liver injury [41]. Overall, similar to CD36, FABP1 overexpression can lead to abnormal liver functions such as dyslipidemia and hepatic steatosis.

## 4. Lipid Abnormalities in the Liver

Abnormal lipid accumulation in the liver is the pathophysiological feature of NAFLD [43]. Lipid abnormalities are of many types. This study mainly focuses on the FA-induced hepatotoxicity, arachidonic acid metabolism and inflammation, and hepatic ceramide overload and hepatic injury.

### 4.1. FA Accumulation and Hepatotoxicity

Lipotoxicity is defined as abnormal cellular lipid composition that leads to the accumulation of toxic lipids, organelle dysfunction, cellular damage, and chronic inflammation. In this study, we focused on the relationship between FA toxicity and NAFLD as well as lipotoxicity, which causes direct damage to mitochondria and peroxisomes [44, 45]. Mitochondrial damage results in the loss of membrane polarization, rendering mitochondria incapable of effectively completing *β*-oxidation and energy metabolism, hence further aggravating FA accumulation. FA exacerbates insulin resistance and promotes inflammation [46–50] and fibrosis [51–53] in progressively worsening liver cells, resulting in liver cell damage. Damaged liver cells release many inflammatory mediators. Inflammasomes, cytokines, chemokines and their receptors, and innate and adaptive immunity cells are all induced by liver inflammation and the direction of NASH treatment [46–49]. Liver inflammation results in the inactivation of tissue repair mechanisms. These mechanisms replace damaged liver cells by activating the staminal compartment, stimulating hepatocyte proliferation, and remodeling the extracellular matrix (ECM) [54, 55]. If inflammation persists for a longer period of time, ECM-forming cells are recruited and activated, leading to excessive deposition of ECM and eventually liver fibrosis [56]. Briefly, FA accumulation causes mitochondrial damage that further aggravates FA accumulation and results in insulin resistance and, finally, liver inflammation and fibrosis.

### 4.2. Arachidonic Acid Metabolism and Inflammation

An elevated level of FFA in the liver is recognized as the leading cause of cell damage and death in NASH [57–60]. A recent study found that although the total lipid content increased, the liver FFA content remained unchanged in NAFLD patients. However, the FFA level in circulation may not be associated with that in cells. This may be because polyunsaturated FAs (PUFAs) play a key role, either proinflammatory or anti-inflammatory depending on their structure, in NAFLD progression [61]. Arachidonic acid is one of the long-chain polyunsaturated omega-6 FAs (n-6 PUFAs), which are the precursors of the potent proinflammatory eicosanoids [62, 63]. The progression from simple hepatic steatosis to NASH may be due to escalation of inflammation [64]. Histologically, both isolated steatosis and NASH present with intracellular lipid accumulation and lipid droplet (LD) formation in the cytoplasm of hepatocytes; however, no inflammation is observed in isolated steatosis, and pathological inflammation resulting in cell necrosis is observed in NASH. LDs are thought to be the source of overproduction of proinflammatory eicosanoids, presenting early involvement of arachidonic acid metabolites in NAFLD [65]. In addition, studies with a lipidomics approach on NAFLD patients have consistently demonstrated that a higher n-6 : n-3 ratio in the blood and liver is associated with the presence and severity of NAFLD [61]. Another study used this approach to quantify the major lipid species in the liver [66] and provide some novel and interesting insights into the pathophysiology of NAFLD. An increase in the level of arachidonic acid and a decrease in the level of key n-3 FAs cause the ratio of n-6 : n-3 FAs to increase. After, arachidonic acid (20 : 4n-6) is released from membrane phospholipids by phospholipase A2 and from the breakdown of phosphatidylinositol bisphosphate through diacylglycerol (DAG) by phospholipase C; it is promptly transformed into proinflammatory prostaglandins, thromboxanes, and leukotrienes by cyclooxygenase [67]. CYP4A14 is a hydroxylase that catalyzes omega-hydroxylation of medium-chain FAs and arachidonic acid in mice and is highly expressed in the liver. In both NAFLD patients and mouse models, CYP4A14 was reported to be significantly upregulated. In addition, CYP4A14 overexpression resulted in increased hepatic lipid accumulation in wild-type mice, whereas CYP4A14 ablation prevented NASH progression [68]. In conclusion, arachidonic acid induces inflammation and, thus, plays an important role in NAFLD.

### 4.3. Hepatic Ceramide Overload and Hepatic Injury

Ceramide, composed of an amino group of a sphingoid base, usually sphingosine and saturated or monounsaturated fatty acyl chains, forms the hydrophobic core of all the complex sphingolipids (sphingomyelin, cerebral gangliosides, and gangliosides) [57]. Ceramide is involved in key steps of the pathogenesis of NAFLD, including the disruption of insulin sensitivity and mitochondrial metabolism, metabolic disturbance, and stimulation of cell death [69–72]. It has been confirmed that the level of hepatic ceramide is elevated in NAFLD and associated with the severity of liver disease [73–75]. Hepatic ceramide overload is caused by an increase in hydrolysis of sphingomyelin through acid sphingomyelinase (ASM). Several studies have reported that elevated levels of sphingolipid in the liver and plasma were consistent with the progress of hepatic insulin resistance, hepatic dysfunction, and steatosis in rodents [76, 77]. Generally, serine palmitoyl transferase (SPT) stimulates the binding of palmitoyl-CoA to serine to form sphingolipids. However, SPT can stimulate the binding of acyl-CoAs to amino acids to produce a group of atypical sphingoid bases such as 1-deoxysphingolipids. Sphingolipids, especially 1-deoxysphingolipids, were regarded as biomarkers of NAFLD progression in a recent omics approach [78]. Myostatin, an SPT inhibitor, reduced ceramide levels in the experimental models of NAFLD [79, 80]. In addition, ASM is associated with the process of simple steatosis to NASH. ASM mRNA levels are three-fold higher in NASH patients than those in healthy controls [81]. Moreover, ASM knockout mice are protected from diet-induced steatosis [82] and NASH [83]. The ASM activity is enhanced in NASH stimulated by proinflammatory substances such as TNF-*α*, reactive oxygen species (ROS), and death receptor ligands [84] and by increased SFAs, which are the key substrates for the de novo synthesis of ceramides. Mitochondria are the main cellular target of ceramide, which damages FA *β*-oxidation, promotes ROS production, TG accumulation, and insulin resistance [85, 86]. Inhibitors such as myriocin (SPT inhibitor) and fenretinide (inhibitor of the enzyme catalyzing DES1 synthesis [last step of bioactive ceramide synthesis]) have been found to ameliorate insulin resistance in experimental models of NAFLD [80, 87, 88]. In all these experiments, inhibition of ceramide synthesis was associated with reduction of hepatic steatosis [89]. Other mechanisms of ceramide lipotoxicity in NASH are as follows: imbalance of calcium homeostasis in the endoplasmic reticulum (ER), leading to ER stress-mediated apoptosis; activation of NLRP3 inflammasome, leading to autophagy damage; and increase in hepcidin expression, leading to liver iron overload [83, 90–93]. In addition, ASM activation may promote liver damage by interfering with the metabolism of methionine and phosphatidylcholine and, thus, contribute to permeabilization of the lysosomal membrane [83] and activation of hepatic stellate cells (HSCs) [81]. Moreover, ceramide has been reported to mediate many of the adverse effects of SFAs, especially palmitic acid, which is a substrate in the SPT response [71, 94]. However, weight loss was reported to reduce steatosis and hepatocellular damage and remarkably alter the expression of ceramide-related genes in the liver. Changes in calorie intake and fat consumption, particularly saturated fat, dramatically correlated with changes in the expression of ceramide-related genes [95]. Briefly, ceramide causes disruption of insulin sensitivity and mitochondrial metabolism, imbalance of calcium homeostasis in the ER, and ultimately liver injury.

## 5. Therapy

### 5.1. Lifestyle Changes and Medication

A strict diet control seems to be an attractive and safe method for treating NASH. Both the Mediterranean and ketogenic diets advocate a reduced intake of carbohydrates. In addition, more physical exercise routinely can prevent and relieve NAFLD by improving lipid homeostasis. Further, statins (lipid-lowering drugs) present some benefits for the liver.

The Mediterranean diet is characterized by decreased intake of sugars and refined carbohydrates and enhanced intake of monounsaturated and n-3 FAs [96]. Different types of lipids (e.g., n-3 and n-6) may have opposite net influence on inflammation, and therefore, the final biological net effect is determined by their relative proportion [57]. Unlike n-6, n-3 PUFAs have important anti-inflammatory effects; they reduce adipogenesis and increase fatty acid oxidation (FAO), leading to a decrease in hepatic steatosis [97–99]. Fish and n-3 PUFA intake are reported to be lower in NAFLD patients than in nonfatty liver patients. Similarly, a downward trend of n-3 FA, eicosapentaenoic acid (20 : 5n-3) and docosahexaenoic (22 : 6n-3), intake is observed in these patients considering multiple lipids [98, 99]. These two n-3 FAs have significant antiproliferative, anti-inflammatory, and modulatory effects on the metabolic and immune systems [57]. The observed decreasing trends in the intake of these key n-3 FAs may promote steatosis, inflammation, dyslipidemia, cell damage, and carcinogenic risk in NASH patients [100, 101]. This reveals the theoretical basis for NASH treatment with n-3 FAs [73]. To date, most studies have used the methods of complementing patients with n-3 PUFAs. In 2012, a meta-analysis of nine studies revealed that n-3 PUFA supplementation has a beneficial influence on the liver fat and liver enzyme level [102]. The Mediterranean diet results in reduced calories, which is acceptable by patients and should be encouraged.

Ketogenic diets (KDs) are very low in carbohydrates and high in fats and/or proteins compared with various diets and, therefore, have gained popularity [103]. Low-calorie, especially low-carbohydrate, KD quickly reduces liver fat content and related metabolic abnormalities [104]. KD has been reported to promote weight loss, reduce intrahepatic triglyceride content, and alleviate metabolic parameters in obese patients. In addition, KD was reported to provoke weight loss in rodents. However, maintaining a long-term KD stimulated the progression of NAFLD and systemic glucose intolerance in mice. Thus, the relationship between KD and systemic insulin resistance in humans and rodents remains to be elucidated [105].

Physical activity (PA) is an integral part of any therapeutic strategy for weight loss, and it may play an important role in preventing NAFLD [106–108]. Multiple cohort studies revealed that change in body weight was correlated with both the development and remission of NAFLD [109, 110]. In men, initiation of an exercise regimen was remarkably associated with NAFLD remission [111]. The role of PA in delaying NAFLD progression has been demonstrated to be beneficial in the presence and absence of one or more metabolic syndromes [112]. In addition, an increase in PA prevents and/or retards NAFLD-related disease progression independent of weight loss [113, 114]. Dietary counseling and appropriate exercise should be combined and adjusted according to individual circumstances, targeting a gradual weight loss of 7%–10% [115, 116].

Atorvastatin and rosuvastatin [117] are widely used for treating dyslipidemia; however, they have not been well established as specific treatments for NAFLD. The guidelines of the European Association for the Study of the Liver (EASL)/European Association for the Study of Diabetes (EASD)/European Association for the Study of Obesity (EASO) [106] consider that statins have not been thoroughly tested. However, a large number of animal and human studies have demonstrated that the use of statins was safe in NAFLD, without an increase in the risk of hepatotoxicity, and may even significantly reduce aminotransferases. Animal data suggested that statins had certain beneficial effects on liver histology in NASH models [118]. Three post hoc analyses of randomized controlled trials in humans revealed that the use of atorvastatin had a beneficial effect on NAFLD in terms of liver enzyme reduction and ultrasonography improvement [118]. Statins may be the valuable option to be considered in patients with NAFLD/NASH, as it significantly reduces the risk of cardiovascular disease and liver cancer simultaneously [119–148].

### 5.2. Targeted Therapy

NAFLD is characterized by ectopic toxic lipid accumulation, which is due to an extensive derangement in hepatic lipid metabolism [149–151]. Underlying these abnormalities is a wide range of disorder of nuclear transcription factors that adjust lipid metabolism, inflammation, and fibrogenesis, which consist of CD36; peroxisome proliferator-activated receptor- (PPAR-) *α*, PPAR-*δ*, and PPAR-*γ*; farnesoid X receptor (FXR); and sterol regulatory element binding protein 1 (SREBP-1), which are ideal targets for NAFLD treatment [152, 153].

CD36 is a FA receptor that plays a significant role in regulating lipid and glucose use, and the upregulation of CD36 expression is associated with NASH [154]. The RNA expression of nuclear factor kappa-B (NF-*κ*B), a key regulator involved in the inflammation process, can be affected by the manipulation of CD36 expression [155]. Several studies have indicated that abnormal expression of CD36 in the liver was markedly associated with insulin resistance, hyperinsulinemia, and steatosis in NAFLD patients [154]. CD36 expression was reported to be elevated in mouse models with genetic obesity and high-fat diet- (HFD-) induced fatty livers [37]. Hence, treatment strategies designed to reverse this process by restoring normal levels of CD36 may provide a new method for treating NAFLD. A study reported that the absence of CD36 in the liver remarkably retarded the development of hepatic steatosis, although FA level increased. In addition, CD36 deletion affected the blood FA composition and improved the serum markers of hepatic inflammation [156]. Further, hepatocyte-specific loss of CD36 was found to significantly improve whole body insulin sensitivity in HFD-fed mice [156]. Briefly, CD36 not only is a disease marker but also plays an active role in FA uptake and has a significant influence on insulin sensitivity and hepatic lipid content and composition [157].

PPAR-*α*, a transcription factor, is mainly expressed in metabolically active tissues and regulates FAO. Lipid accumulation owing to FAO inhibition indirectly accelerates fibrogenesis by promoting inflammation. Agonists of PPAR-*α* presented beneficial effects of reversing deficiencies in FAO and improving NAFLD progression in animal and cell models [157, 158]. A randomized controlled trial demonstrated that pharmacological modulation of the PPAR-*α* nuclear receptor leads to substantial histological improvements in NASH patients, including the improvement of steatohepatitis and alleviation of cardiometabolic risk profile, with a sound security profile [157]. Several studies have emphasized on developing dual agonists against PPAR-*α* and PPAR-*δ*. The effects of PPAR-*α* promoted FAO [158], and those of PPAR-*δ* reduced de novo lipogenesis and inhibited inflammation [159, 160]. One such agent, elafibranor (GFT505), improved diet-induced NASH in rodents [160]. Elafibranor has been reported to improve hepatic and peripheral insulin sensitivity in humans [161]. In addition, improvements in NASH, insulin resistance, and dyslipidemia to some extent have been reported in long-term studies [157]. In summary, PPAR-*α* is important in reversing deficiencies in FAO and alleviating NAFLD progression.

The most widely studied drugs with potential benefits for NASH are thiazolidinediones (TZDs). TZDs such as pioglitazone and rosiglitazone activate the nuclear receptor, PPAR-*γ*, allowing preadipocyte differentiation into insulin-sensitive, fat-storing adipocytes [106]. It is noteworthy that the PPAR-*γ* ligands attenuate liver fibrosis by inhibiting transdifferentiation of liver stellate cells into activated myofibroblasts, suggesting a direct hepatoprotective influence. In addition, they present anti-inflammatory effects and increase circulating adiponectin, which is an adipokine that resists adipogenesis, and insulin sensitization [106]. Similarly, a clinical research study reported that they ameliorate glycemic control and NASH-associated parameters [162–164]. However, rosiglitazone has been withdrawn from the market in most of the countries due to the deficiency and the possible long-term treatment. We hold the opinion that it is necessary to overcome this obstacle [108, 117]. According to the current guidelines, pioglitazone is useful and is advocated for elderly patients with advanced fibrosis, confirmed by biopsy, who are unable to adopt or maintain lifestyle interventions and have persistent metabolic risk factors; however, pioglitazone should be administered to patients with T2DM and/or heart failure with caution [162, 165, 166]. Meta-analyses have revealed that rosiglitazone and pioglitazone were remarkably better than placebo in relieving balloon formation, lobular inflammation, and steatosis [167, 168]. Pharmacological inhibition of PPAR-*γ* resulted in the amelioration of NAFLD development [169]. For instance, in HFD-fed mice, hepatocyte/macrophage-specific PPAR-*γ* knockout protected against hepatic steatosis and PPAR-*γ* knockdown induced by RNA interfering-adenovirus vector injection improved a fatty liver [10, 170, 171]. In summary, TZDs improved hepatic steatosis and alleviated NASH and liver fibrosis by increasing insulin sensitivity in skeletal muscle and adipose tissue, thus overcoming the direct steatogenic influence on liver cells [171–173].

Bile acids (BAs) and their receptors (e.g., BA nuclear receptor and FXR) play indispensable roles in regulating systemic metabolism and hepatic lipid homeostasis. FXR is a nutrient-sensing nuclear receptor in the gut and liver that regulates glucose and fat metabolism [174]. These functions of FXR were assessed through a quantitative proteomic analysis of mouse liver tissue [175]. Studies had found that FXR regulates amino acid catabolism and detoxification of ammonium in the livers of mice through ureagenesis and glutamine synthesis. Further, the synthesis of ceramide pools throughout the body was reported to be regulated by the BA/FXR axis in the ileum and cecum [176]. Intestinal specific genetic or pharmacological inhibition of FXR led to a decrease in circulating ceramide levels, increase in browning of adipose tissue, and amelioration of liver insulin resistance and liver injury in HFD-induced obese mice [177]. Activation of hepatic FXR has been shown to reduce liver glucose, adipogenesis, and steatosis in animal models [178]. FXR is important for liver inflammation and has been proven to be a potential therapeutic target for NASH [179, 180]. FXR activation reprograms arachidonate metabolism in mice [181] and stimulates 1-deoxysphengolipid catabolism and thereby attenuates the cytotoxic effects [182]. A phase 2 randomized double-blind placebo control trial in Japan showed that compared with placebo, high doses of obeticholic acid (OCA) intake remarkably resolved NASH [178]. In the FXR Ligand OCA in NASH Treatment (FLINT) trial, OCA induced NASH remission in 22% of patients, whereas placebo induced NASH remission in 13% of patients [157]. In summary, FXR regulates circulating ceramide levels and improves hepatic insulin resistance and liver damage in NAFLD/NASH patients.

Hepatic de novo lipogenesis is stimulated by activation of the nutrient-sensing mTorc1 pathway, a substrate of insulin-Akt signaling under physiological conditions [179]. For example, treatment of hepatocytes with rapamycin, an allosteric inhibitor of mTorc1, inhibited insulin activation of the lipogenic transcription factor SREBP-1c [183, 184]. In addition, specific knockout of the mTorc1-defining component, raptor, in the liver alleviated HFD-induced hepatic steatosis, which may be due to reduced lipogenesis [185]. It has been demonstrated that Notch antagonism uncouples Akt from mTor activation, implying that NAFLD can be treated by Notch antagonists [186].

## 6. Conclusion

This article documented recent advances in lipid abnormalities in NAFLD. We proposed that an abnormality in lipid metabolic pathways eventually leads to NAFLD. This viewpoint was theoretically and experimentally validated by elaborately elucidating the role of FA, arachidonic acid metabolic disorders, and ceramide overload in the pathogenesis of NAFLD. In addition, we offered some treatment options for NAFLD. All these observations and experimental findings provide the scientific basis for the prevention and treatment of NAFLD.
